# Adaptive Hybrid PSO–APF Algorithm for Advanced Path Planning in Next-Generation Autonomous Robots

**DOI:** 10.3390/s25185742

**Published:** 2025-09-15

**Authors:** Abdelmadjid Benmachiche, Makhlouf Derdour, Moustafa Sadek Kahil, Mohamed Chahine Ghanem, Mohamed Deriche

**Affiliations:** 1 Laboratory of Computer Science and Applied Mathematics, Chadli Bendjedid University, El-Tarf 36000, Algeria; 2 Laboratory of Artificial Intelligence and Autonomous Objects, Larbi Ben M’hidi University, Oum El Bouaghi 04000, Algeria; derdour.makhlouf@univ-oeb.dz (M.D.); moustafa.kahil@univ-oeb.dz (M.S.K.); 3 School of Computer Science and Informatics, University of Liverpool, Liverpool L69 3BX, UK; 4 School of Computing and Digital Media, London Metropolitan University, London N7 8DB, UK; 5 Artificial Intelligence Research Centre, College of Engineering and Information Technology, Ajman University, Ajman P.O. Box 346, United Arab Emirates; m.deriche@ajman.ac.ae

**Keywords:** PSO, APF, navigation, autonomous mobile robot, obstacle avoidance, path planning

## Abstract

The field of autonomous robotics is progressing rapidly, with research moving toward developing systems capable of moving without direct human control and learning without human intervention. One of the problems requiring an efficient and sustainable solution is ensuring the smooth and safe navigation of robots between obstacles. In this study, a new path planning approach is developed, integrating particle swarm optimization (PSO) and artificial potential field (APF) algorithms to assist the mobile robot in navigating an area with static and dynamic obstacles. The robot moves independently while routing dynamically and avoiding obstacles. To evaluate its adaptive ability to a changing environment, we continuously calculate the shortest distance between two points and dynamically adjust the path to avoid obstacles during replanning, path recalculation, and robot position adjustment to ensure efficient and safe navigation. Different scenarios are tested to evaluate our approach, including different environmental conditions and obstacle configurations. Experimental results show that our method reduces the path length by 18%, the obstacle avoidance efficiency by 90%, and the success rate by 85% in dynamic environments. In addition, PSO-APF reduces computation time, demonstrating better capacity and efficiency.

## 1. Introduction

Autonomous mobile robotics focuses on creating systems that can independently navigate and engage with their surroundings without needing direct human control. These robots are designed for operation in dynamic and unpredictable environments, such as warehouses, factories, outdoors, and disaster areas. The main goal is to allow robots to perform tasks autonomously while reducing the demand for human intervention [1,2]. The global market for autonomous mobile robots is projected to surpass USD 30 billion by 2030, mainly fueled by uses in logistics, disaster response, and healthcare. For example, Business Insider (published on 11 February 2025) reported that Amazon employs more than 750,000 robots in its warehouses, which facilitate automation for approximately 75% of its global package deliveries. Despite this remarkable progress, navigation mistakes caused by environmental uncertainties and moving obstacles still occur, often resulting in errors in complex real-world situations. These developments underscore the urgent need for robust and adaptable path-planning algorithms that can deliver dependable results in diverse and dynamic settings.

The diverse challenges of real-world environments emphasize the importance of autonomous navigation. Unlike controlled lab or industrial spaces, real-world areas are marked by unpredictability and ongoing change. Autonomous robots must grapple with challenges such as diverse obstacles, fluctuating terrains, dynamic entities (such as moving objects or individuals), and uncertainties in sensor data and environmental conditions [2]. What drives us to explore autonomous robotic navigation is to bridge the gap between current technological capabilities and the multiple requirements of real-world applications. There are still significant barriers to achieving substantial progress in robotics, requiring further developments to enable reliable and efficient operation in complex environments [3].

Despite the efficiency of industrial robots at performing repetitive tasks in controlled environments, they face limitations when operating in unfamiliar settings or under unexpected conditions. Their rigid programming restricts their flexibility to cope with dynamic changes within their environment; they are limited to operating flexibility and autonomy and, in most cases, work within predetermined sequences of actions in highly tuned environments [1,4]. A clear trend is emerging with rising demand for genuinely autonomous robots that can operate independently. These robots are expected to possess the intelligence and flexibility necessary to navigate complex environments, make swift decisions, and perform tasks without ongoing human supervision. This increasing demand emphasizes the recognized benefits of autonomous systems in boosting productivity, efficiency, and safety across various industries and applications. Consequently, there is a distinct move toward developing robots with advanced sensing, perception, and decision-making capabilities to meet the evolving needs of modern industries and societies [4].

Autonomous navigation in complex, dynamic environments remains a significant challenge in robotics and intelligent systems, where conventional techniques, such as Artificial Potential Fields (APFs), are straightforward to implement and require minimal computing power. However, they frequently face issues like becoming stuck in local minima and oscillations, particularly in dense environments filled with obstacles. Conversely, global optimization techniques like particle swarm optimization (PSO) are effective at exploring solutions but can be slow and occasionally generate suboptimal paths in large-scale navigation problems. These challenges underscore the importance of hybrid approaches that integrate the rapid response capabilities of local planners with the extensive search capabilities of metaheuristic techniques. The field of multi-agent systems faces analogous hurdles, where protocols for Fast Finite-Time Consensus (FFTC) in high-order nonlinear systems leverage event-triggered communication to reduce computational burden and enhance adaptability in dynamic environments [5]. While such methods excel in achieving consensus under adversarial conditions, this paper presents the PSO-APF framework, which offers a complementary strength: real-time, adaptive parameter tuning for robust navigation and obstacle avoidance in unpredictable settings. Unlike traditional hybrid methods, which depend on preset or offline-tuned parameters, this approach features an adaptive layer. This adaptivity includes real-time optimization of APF parameters (η, $d_{0}$, *w*, $c_{1}$, $c_{2}$) through PSO during navigation. This dynamic adjustment allows the robot to repeatedly reconfigure its force field in response to environmental changes, enhancing its responsiveness, robustness, and ability to escape local minima.

This research aims to address the challenge of developing an autonomous mobile robot capable of navigating an uninterrupted path from an origin to a destination. The navigation and positioning of the robot reveal two main perspectives: the first is to direct the robot from a starting position to a destination, while the second is to choose the best path from the robot’s current location. We aim to confirm that the combined use of PSO and APF techniques can effectively address both navigation and positioning problems, providing satisfactory solutions. PSO is a bio-inspired algorithm that shows promise for optimization techniques inspired by natural swarm behavior. On the other hand, APF techniques utilize virtual forces to enable the robot to navigate around obstacles. Bio-inspired algorithms, such as PSO, are rapidly emerging as viable solutions to optimization issues. PSO achieves global optimization through iterative improvement of candidate solutions; hence, it is a valuable approach for both industry and academia. Similarly, the interest of researchers has extended to the topic of the APF due to its potential future applications in collision avoidance by robots, which employ virtual forces. We propose a new method that combines PSO with APF to overcome the limitations of each technique. While the PSO technique may struggle to operate in high-dimensional spaces, APF techniques can encounter problems such as becoming trapped in local minima; hence, our approach aims to leverage the strengths of both while mitigating their drawbacks.

In summary, traditional path-planning methods are effective but often lack flexibility and robustness in dynamic, uncertain environments. This research proposes a solution by integrating PSO with APF, resulting in an algorithm that leverages PSO’s global search capabilities alongside APF’s real-time adaptability. The main contributions of this paper can be summarized as follows:

- We developed a new adaptive hybrid PSO-APF algorithm that allows PSO to dynamically tune APF parameters (repulsive scaling η and distance of influence $d_{0}$) in a way that favors global optimization while also providing rapid tuning in the local scope.
- In our experiments with dynamically changing scenarios, the suggested algorithm provides better navigation performance, with significantly shorter paths (up to 18%) and an 85% success rate, relative to PSO and APF alone representations, utilizing moving obstacles, in comparison with two standalone benchmarks and one hybrid benchmark.
- A thorough review of parameter sensitivity and parameter optimization was performed, which provides a systematic exploration of how basic parameter choices can affect performance, along with practical advice on parameter knobs to tune.
- The proposed adaptive hybrid PSO-APF algorithm is robust and flexible in various contexts by exploiting real-time replanning to consider dynamic or moving obstacles with degraded paths, as well as dynamic or different goals, which all reflect reliability in a real-world implementation scenario.

The rest of the paper is organized as follows. Section 2 reviews related work on both global and local path-planning methods. Section 3 describes the main components of PSO and APF and discusses their relevance to some aspects of reinforcement learning and adaptive control methods. Section 4 details the PSO-APF hybrid system we propose, including its initialization, how we evaluate fitness, and how we adapt the PSO-APF scheme during operation. Section 5 presents simulation results with comparisons of performance metrics. Finally, Section 6 concludes the paper, discussing future research directions, such as assistive robots, and considerations for integrating an APF into a sensor-fusion framework.

## 2. Related Work

In this section, we will explore diverse methodologies adopted by researchers to solve navigation challenges faced by autonomous mobile robots. Mobile and autonomous robotic navigation is the ability to move independently in dynamic environments filled with obstacles, achieved by using information collection and decision-making techniques without human intervention [6]. Navigation, being a pivotal task in mobile robotics, can be broadly categorized into two types: global navigation and local navigation [7,8]. When the path is determined from the starting point to the destination, this is called global path planning, while local path planning is modifying the path to avoid potential hazards and correcting the path in real time [9]. Table 1 presents a comparison between global and local path planning. Additionally, we will examine integrated navigation strategies and hybrid reinforcement learning–PSO approaches.

### 2.1. Global Path Planning

This navigation utilizes pre-existing environmental data to calculate the optimal path from start to destination before the robot begins moving, thereby determining the best and most efficient route. Several techniques have been developed, including the Dijkstra algorithm, Floyd–Warshall algorithm, Bellman–Ford algorithm, A* algorithm, deep learning, genetic algorithm (GA) [8], Bacterial Foraging Optimization (BFO) [10], PSO, ABC, Ant Colony Optimization (ACO), Golden Jackal Optimization, and Grey Wolf Optimization. The efficient global path planning method PSO helps robots navigate efficiently by optimizing the route using a swarm of particles that search for the best path based on a set of evaluation criteria [8]. Researchers have developed numerous innovative PSO variants, including PSO-GA, an efficient algorithm for large-scale global path planning of UAVs. It is a hybrid algorithm that combines GA and PSO to find the optimal path in a grid environment. It optimizes paths considering 4G network coverage, communication reliability, power constraints, obstacles, and no-fly zones. PSO-GA shows faster convergence and better performance in suburban environments, validated by real-world tests [11]. BAPFACO is a novel global path-planning algorithm that improves ACO. This technique integrates a bidirectional APF to eliminate traps, an adaptive heuristic function to reduce turns, a pseudo-random state transition rule to accelerate convergence, and an improved pheromone update strategy to escape local optima. BAPFACO outperforms other ACOs by reducing path length, improving convergence speed, and minimizing turning times [12]. To effectively optimize the path planning of a mobile robot with global optimization, improve the path smoothness, and reduce running time, Huang et al. [13] used the A* PSO (APSO) technique, which combines A* and PSO. Researchers in another article (Ref. [14]) proposed SDPSO, a novel approach for efficient UAV global path in complex environments. SDPSO enhances global optimization and reduces local convergence, outperforming traditional methods by improving path quality and robustness in 3D environments. Lv and Yang [15] improved PSO for mobile robot path planning, where this method features an inverse learning strategy that initializes the swarm and dynamically adjusts the inertial weight and learning factors during iterations to improve the global search capability, convergence accuracy, and stability. The authors in this approach utilize the Travelling Salesman Problem (TSP) to frame the path planning, and PSO to optimize this path. Zhong and his team [12] proposed an alternative global path planning algorithm to optimize the mobility efficiency of robots, which generates paths segmented by turning points and smooths them using Dubins and Clothoids curves under kinematic constraints. They used an evaluation function to select the most efficient path considering factors such as speed loss and turning distance. The results demonstrate the effectiveness of this method, which outperforms the Hybrid A* algorithm in generating smooth and efficient trajectories in complex environments. Researchers [16] introduce an adaptive PSO that enables smooth path planning in mobile robots and avoids obstacles. They adjust the inertial weight and acceleration coefficients according to the swarm’s success rate in the current iteration to enable dynamic adaptation in the search space. To streamline multi-UAV collaboration, the authors in [17] used the PPSwarm algorithm that combines RRT* for fast initial path search and a priority-based method. PPSwarm outperforms other algorithms in terms of path quality, speed, scalability, and scenarios with up to 500 UAVs. ABC-PSO is primarily a global path planning algorithm, as it seeks to find the optimal path by searching the entire space, and enhances the global search capabilities of PSO by integrating the global and local search mechanisms of the artificial bee colony (ABC). This makes it suitable for global navigation missions [18]. HWPSO is a hybrid algorithm that combines the Whale Optimization Algorithm (WOA) and PSO for global path planning of differential wheeled mobile robots, aiming to solve the challenges of nonholonomic constraints in complex terrains and to exploit the exploration capabilities of WOA and the exploitation efficiency of PSO. Najm et al. [19] evaluated HWPSO in five different environments and compared it with several works, where the results show its effectiveness. The authors of another study [20] presented an improved PSO for mobile robot path planning. They address the low convergence accuracy and premature convergence. To improve the search accuracy, they integrate differential evolution (DE) with adaptive parameters and a “high-intensity training” mode. To optimize the path length and hazard level, they used a two-objective approach. Separately, Haris and Nam [21] have sought to utilize distance-dependent sigmoid inertia weighting (DSI-PSO), a novel technique for enhancing route planning. It aims to find an optimal and collision-free path that satisfies criteria such as shortest distance and smoothness, which are characteristic of global planning, the authors of DSI-PSO inspired by the activation functions of neural networks, where it adapts its inertia weight using a sigmoid function based on the distance metric of a particle, DSI-PSO shows high performance in convergence rates and path optimization, including safe and efficient route planning. Makhlouf et al. [22] combined PSO and BFO to find the optimal path from a starting point to a destination in obstacle-ridden environments. This approach is tested in various scenarios and demonstrates its effectiveness in solving path planning problems. The authors introduce an enhanced deep reinforcement learning algorithm for global path planning of autonomous mobile robots (AMRs) in complex, unfamiliar environments. This method treats the path-planning challenge as a Markov Decision Process (MDP). It employs a Double Deep Q-Network (DDQN) along with an adaptive ϵ-greedy strategy and a heuristic-based reward system to promote efficient exploration and successful goal achievement. The approach produces safe, concise, and smooth paths by selecting optimal actions and applying Bezier curve smoothing. Simulation results indicate that the proposed IDDQN algorithm surpasses traditional methods such as DQN, A*, RRT, and APF in path efficiency, safety, and disturbance resilience, showing strong adaptability in unfamiliar settings [23].

### 2.2. Local Path Planning

Local path planning enables robots to determine how to move and operate in unfamiliar or changing environments, utilizing real-time data from sensors to continuously update the path, distance to the target, and obstacle positions while navigating. Katona et al. [8] have successfully employed a variety of methods such as Bug algorithms, APF, Rapidly Exploring Random Tree (RRT), Simulated Annealing, and Tabu Search. To address the challenges posed by local navigation, several studies have followed this approach, including the AS-IAPF (Adaptive Strength Improved APF). This was presented to address congestion, obstacle avoidance, and path planning efficiency of autonomous vehicles in urban environments. The scholars [24] combined a dynamic traffic stability model with improvements in the APF algorithm, including Gaussian oscillation for path optimization and adaptive repulsion force adjustments. The AS-IAPF is efficient and stable, outperforms traditional APF methods, and improves safety through 2D and 3D scenario simulations. The ISALPSO (Improved Self-Adaptive PSO) algorithm is used to enhance the path prediction of mobile robots in logistics applications. Fusic and his colleagues [25] used 2D Lidar mapping and the Hector SLAM method on the Robot Operating System (ROS) platform to create real-time maps and find efficient routes. Simulation and hardware test results demonstrate that ISALPSO is effective in optimizing path planning, yielding shorter trajectories, faster convergence, and reduced computation time compared to other PSO variants. Benmachiche et al. [26] use GA to determine optimal paths for multiple robots to navigate in changing environments. Robots edit their paths based on real-time conditions, and path evaluation is based on a fitness function, which optimizes path length. The authors of another work [27] presented how fuzzy logic and bright lights can help autonomous vehicles navigate their environment. The problems addressed include guiding robots from one point to another and finding the best path. Using the Reinforcement Learning PSO (RLPSO) algorithm, Nagar et al. [28] looked into optimizing path planning for an autonomous ground vehicle (AGV) that navigates in real time through localization and map generation via Hector-SLAM and LiDAR sensors. RLPSO eliminates obstacles and follows feedback, where the results show that RLPSO outperforms other PSOs in both simulation and real-world testing. Muni et al. [29] used the SOMA-PSO algorithm to respond to obstacles detected by sensors while maintaining path efficiency. It combines the self-organizing migration algorithm (SOMA) and PSO for optimal path planning and control of single and multiple mobile robots in static and dynamic environments. In addition to classical optimization-based techniques, several modern control approaches have been applied to robot local path navigation. For instance, the researchers in this study present an improved local path planning algorithm called FLC-TEB for car-like mobile robots. It builds on the Timed Elastic Band (TEB) method by adding objectives for trajectory smoothness and minimal jerk. A -PSOler adaptively modifies the importance of these goals depending on environmental constriction and turn complexity. This strategy yields trajectories that are smoother, shorter, and more stable than those generated by the standard TEB algorithm, as demonstrated through simulations and real-world tests [30]. Kumar et al. present a Deep Spiking Q-Network (DSQN) designed for mobile robot path planning that is both efficient and energy-conserving. This approach replaces the typical neurons in a Deep Q-Network (DQN) with leaky integrate-and-fire (LIF) spiking neurons, which are inspired by biological systems, allowing the DSQN to learn its navigation strategy through reinforcement learning. The DSQN system combines the robot’s overall objectives with local environment observations (such as nearby obstacles) into a single framework, allowing the robot to make intelligent, real-time decisions [31].

### 2.3. Hybrid Navigation

Some methodologies combine global and local path planning, such as BPPSO, or bi-population PSO, which divides particles into two subpopulations: one improves global search capability by considering particle quality and optimal solutions, and the other improves local search by using an adjustable cognitive coefficient. BPPSO outperforms traditional PSO and other algorithms in terms of path quality and execution time [32]. To help robots navigate around obstacles and reach their destination efficiently, the scholars [33] presented a novel approach that creates a path-planning method for mobile robots. This approach adjusts the robots’ movements in response to real-time environmental changes and determines the shortest path with the optimal cost. The research presents a hybrid system for path planning and correction to assist factory handling robots. It utilizes an enhanced A* algorithm to optimize overall path planning and employs the Dynamic Window Approach (DWA) for local adjustments, thereby combining their strengths. Additionally, a fuzzy PID controller was developed to correct navigation deviations. The results demonstrated that this integrated method increased planning efficiency, supported smooth sliding and obstacle avoidance behaviors, and produced stable, efficient paths [34]. In another study [35], the authors present a detailed navigation framework for an Autonomous Marine Surface Vehicle (AMSV) that integrates multi-objective global path planning with real-time obstacle avoidance. A spiral path planner generates a smooth overall route through multiple waypoints, which is subsequently optimized by an improved A* algorithm to steer clear of obstacles dynamically. To maintain precise path tracking despite environmental disturbances such as waves, the system employs a finite-time Extended State Observer (ESO) to estimate and counteract sideslip angles and external forces. This method, combined with a Line-of-Sight (LOS) guidance law and a high-order sliding mode controller, guarantees robust, precise, and stable trajectory tracking.

### 2.4. Multidisciplinary Navigation Insights

Recent advancements in optimization across multiple fields provide valuable insights for autonomous navigation. Bayesian inferential motion planning using heavy-tailed distributions like Student’s-*t* has been suggested to improve motion planning under uncertainty. Unlike conventional Gaussian sampling, heavy-tailed models increase robustness to outliers and rare events, facilitating more effective exploration of the configuration space and collision avoidance in cluttered environments [36]. This probabilistic methodology encourages further enhancements in path planning resilience amid unpredictable dynamics. In structural optimization, the performance-based design of reinforced concrete wall-frames and steel chevron-braced frames using metaheuristics like ABC and ACO shows how swarm intelligence handles nonlinear, high-dimensional problems [37,38]. These studies demonstrate how optimization can balance multiple performance goals, similar to navigation, where safety, efficiency, and real-time constraints must be simultaneously optimized. For robotic manipulators, a closed-loop approach to direct–indirect optimization has been developed to solve nonlinear optimal path-tracking issues [39]. Unlike purely offline trajectory design, this method incorporates real-time feedback and corrections, thereby improving stability and accuracy, which closely aligns with our adaptive PSO-APF system that dynamically adjusts parameters based on environmental changes. Furthermore, convex optimization-based trajectory planning has become a powerful technique for cooperative robotics [40]. By incorporating global performance metrics into real-time control, convex programming allows for the creation of nearly optimal paths even in changing conditions. This method is similar to our approach of using metaheuristics for local planning to improve responsiveness and reliability in dynamic environments. Overall, these interdisciplinary studies support our key strategies: (1) using probabilistic inference to increase search flexibility; (2) applying swarm intelligence for performance optimization under nonlinear constraints; (3) incorporating closed-loop adaptivity for robustness; and (4) achieving optimality through advanced optimization frameworks. These perspectives position PSO-APF at the crossroads of robust, adaptive, and efficient navigation solutions.

### 2.5. Reinforcement Learning–PSO Hybrid Approaches

Katona et al. [8] and Maysam and Al-Darraji [41] classified path planning algorithms into traditional algorithms, such as Bug algorithms, Vector field histograms, probabilistic cell decomposition, etc., and heuristics, such as A*, ga, PSO Ai algorithms (RL, ANN), etc. The authors focused their paper on the most commonly used AI-based methods and their capabilities to solve path planning problems, including deep learning-based path planning, reinforcement learning-based path planning, and deep reinforcement learning-based path planning. Many other researchers explore these techniques in their papers: BILSTM-PSO-GRRT [42] is a novel approach that aims to improve UAV path planning in complex 3D environments with many obstacles, where GDRRT is an enhanced version of RRT* that uses intelligent sampling based on target distance for faster convergence, PSO is used to optimize the paths found by GDRRT*, and BILSTM is a neural network that estimates optimal paths from PSO-GDRRT* and enables real-time path planning. Liu and others [43] optimize reinforcement learning hyperparameters to improve convergence speed and better learning. They reduce the impact of invalid particles by improving the PSO, and they use reinforcement learning rewards to improve particle fitness in the PSO. RMPSO [44] is a novel approach that combines reinforcement learning and PSO to optimize the path planning algorithm for autonomous underwater vehicles. This approach aims to improve the convergence speed and adaptability of PSO by integrating a reinforcement learning feedback mechanism. To optimize the paths of automated guided vehicles, Chen et al. [45] used two algorithms: APF and double delayed deep deterministic policy gradient (TD3). This approach generates optimal paths and considers obstacles, target points, and AGV states. It uses reinforcement learning to select the best actions. The algorithm proposed by Zhang et al. [46] is a multi-objective PSO algorithm with multi-mode collaboration based on reinforcement learning (MCMOPSO-RL). It utilizes reinforcement learning to select the optimal position for particles, striking a balance between exploration and exploitation, and employing a hybrid update mode to enhance performance and solve the collaborative path planning problem for multiple drones in complex environments. The authors [47] proposed the Reinforcement Learning PSO (RLPSO) algorithm, a combination of Q-learning and PSO. RLPSO utilizes Q-learning to explore the environment and create a Q-matrix, which PSO subsequently employs to enhance path planning.

## 3. Background and Motivation

### 3.1. Mechanics of PSO and APF Integration

The hybridization of PSO and APF combines the global optimization capabilities of PSO with the real-time obstacle avoidance of APF (as summarized in Table 2).

This integration establishes a robust framework that strikes a balance between global optimization and local responsiveness. The interaction between the two methods occurs as follows:

- PSO initializes a population of particles (potential paths) in the search space, evaluating their fitness based on a combined cost function that accounts for path length, smoothness, and goal proximity.
- APF calculates repulsive forces from obstacles and attractive forces toward the goal in real-time. These forces provide local adjustments to particle trajectories, ensuring collision-free navigation.
- At each PSO iteration: The APF module provides feedback by modifying the velocity vector of each particle to account for dynamic obstacles. This refined trajectory is then evaluated by the PSO fitness function, integrating local obstacle avoidance into the global optimization process.
- The search space is dynamically adjusted by APF forces, effectively steering particles away from infeasible regions while maintaining focus on the global objective.

The hybrid APF-PSO approach combines the strengths of both methods while addressing their individual weaknesses, making it an effective solution for path planning in complex and dynamic environments (Table 3).

- Weaknesses addressed:
  – While PSO is powerful for global optimization, it can be slow due to its iterative nature. In the hybrid approach, PSO is used for initial path planning, which is completed in advance. This enables the faster APF component to handle modifications in real-time, ensuring rapid responses during execution.
  – APF can get stuck at a local minimum, preventing it from finding the target. The hybrid approach avoids this by using PSO to generate a globally optimized path, giving the APF a solid starting point and reducing the risk of getting stuck.
  – APF often struggles with smooth motion near obstacles, leading to oscillations. The hybrid approach facilitates these transitions, as the globally optimized path of PSO reduces the sudden adjustments required by APF.
- Enhanced strengths:
  – PSO excels at finding efficient paths in congested environments. This global optimization ensures that the path is not only feasible but also efficient in terms of distance and energy usage.
  – APF is fast and lightweight, making it ideal for responding to sudden changes, such as moving obstacles. In the hybrid approach, APF fine-tunes the path in real time without the need for the more computationally expensive PSO restart.
- Benefits: The hybrid APF-PSO method strikes a balance between optimization and adaptability. As shown in Table 3:
  – It combines the efficient pathfinding of PSO with the real-time adjustment ability of APF, ensuring successful navigation even in dynamic environments.
  – Smoother trajectories: By combining global planning with local adjustments, the resulting trajectories become less steep and more natural.
  – Although it requires more computation than APF alone, the hybrid method remains efficient compared to standalone PSO or other hybrid methods.
  – The hybrid method effectively adapts to changing environments, optimizes the search space for efficiency, and overcomes common challenges such as local minima. This synergy makes it a powerful and reliable choice for real-world robotics applications.

### 3.2. Reinforcement Learning and Adaptive Control: Key Differences

Chaoyang [48] employed reinforcement learning to optimize path planning by learning from past experiences. However, reinforcement learning methods often suffer from high computational complexity and may require extensive training data, which can limit their real-time adaptability. Our Approach: In contrast, we combine PSO (global optimization) with APF (real-time obstacle avoidance), which does not require prior training. This hybrid approach ensures faster convergence and adaptability to dynamic environments without the overhead of model training or extensive computation.

Optimal Fully Actuated System Approach: Tian and his colleagues. Ref. [49] focused on trajectory tracking for fully actuated robotic systems, primarily in structured environments with predefined trajectories. Its scope is more on control strategies than on dynamic path planning in unstructured environments.

Our Approach: Our work addresses explicitly dynamic path planning in unstructured and dynamic environments, where both the goal and obstacles can move unpredictably. The hybrid PSO-APF algorithm ensures that the robot navigates efficiently while adapting to environmental changes in real-time.

The referenced studies focus on specialized methodologies for either learning-based optimization or control mechanisms, while our work uniquely integrates a global metaheuristic (PSO) with a local reactive method (APF) to achieve efficient and adaptable path planning.

### 3.3. Adaptive Parameter Optimization

Several hybrid approaches exist in the literature; for example, PSO-GA [11] combines global exploration (GA) and local refinement (PSO), but relies on fixed parameters that must be manually adjusted for specific environments. Similarly, BFO/PSO [22] integrates bacterial search schemes to improve the exploration capability of PSO, but does not allow dynamic parameter adjustment during execution. Our method is fundamentally distinguished by its adaptive mechanism. Our approach achieves true adaptability by modifying PSO-driven APF parameters in real time, setting it apart from traditional hybrid methods where parameters are generally fixed or optimized offline. More accurately:

- Parameter tuning: PSO dynamically adjusts the repulsive scaling factor (η), influence distance ($d_{0}$), inertial weight (*w*), and acceleration constants ($c_{1},c_{2}$) of the APF during each iteration. In contrast, PSO-GA or BFO/PSO usually depend on fixed static values or offline tuning methods.
- Escaping local minima: While genetic algorithms rely on mutation and BFO on chemotaxis to avoid local minima, our approach combines the repulsive forces of APF with PSO’s global exploration, resulting in more effective trap avoidance.
- Real-time tuning: The proposed PSO-APF continuously updates APF forces during operation, whereas BFO/PSO/PSO-GA hybrids are usually designed for offline optimization and then operate in a fixed configuration.
- Two-way feedback loop: In PSO-APF, a two-way feedback loop exists: PSO sends updated parameters to APF for trajectory planning, and APF guides PSO particles via its force field evaluation. This interaction is absent in traditional hybrid methods.

The adaptive nature of our method allows for better performance in changing environments, where fixed-parameter methods have difficulty keeping optimal navigation behavior.

## 4. The Proposed System

The problem revolves around enabling autonomous robots to navigate from a starting point to a goal location in dynamic environments filled with obstacles. The objective is to find an optimal or near-optimal path that minimizes travel time, distance, or energy consumption while avoiding collisions with obstacles.

The hybrid PSO-APF path-planning problem aims to develop a robust navigation strategy for autonomous robots operating in dynamic environments. By integrating the strengths of both PSO and APF, this approach seeks to overcome the limitations of individual methods and achieve efficient and adaptable path planning.

PSO is a bio-inspired optimization algorithm that mimics the behavior of bird flocks or fish schools in nature. It optimizes solutions iteratively by adjusting the positions of particles in a search space to find the best possible solution. In the context of our path planning, PSO is utilized to generate candidate paths by iteratively exploring the environment. Each particle in the swarm represents a potential solution (a path), and the swarm iteratively adjusts the positions of particles based on their individual experiences and the global best solution found by the swarm.

**Figure 1:**
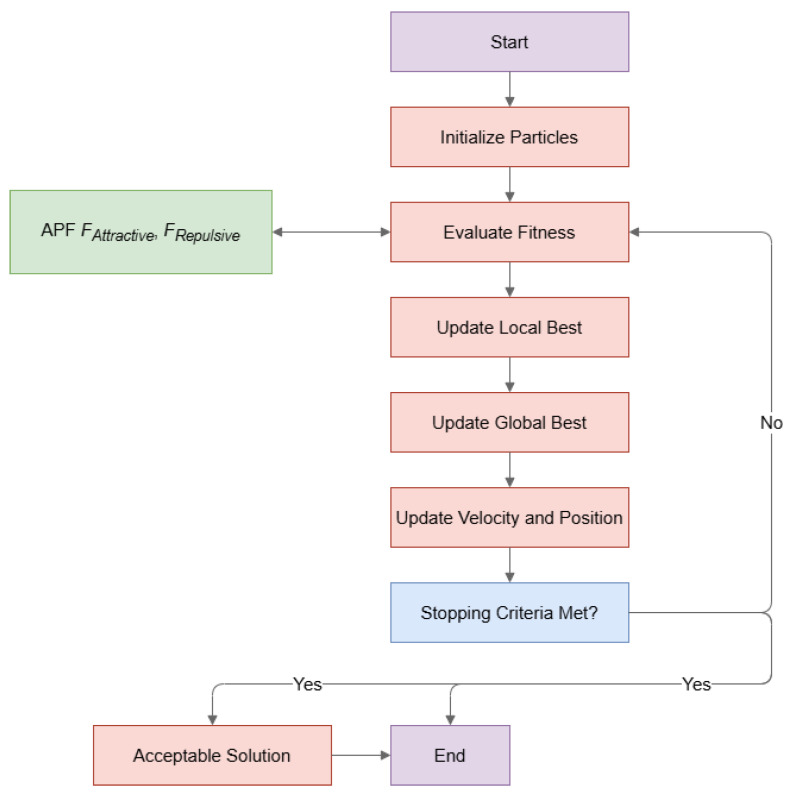
Overall process of PSO-APF.

In contrast, APF operates on the principle of potential fields, perceiving obstacles as repulsive forces and the target destination as an attractive force. Through computation of the potential field surrounding obstacles and the goal, APF influences the robot’s trajectory, diverting it from obstacles and aligning it towards the target. The resultant force dictates both the direction and velocity of the robot’s motion, facilitating progress toward the objective while sidestepping obstacles (Figure 1). Nonetheless, APF may encounter challenges such as being prone to local minima, navigating through narrow passages, or experiencing oscillations around obstacles when used in isolation.

Through the fusion of PSO and APF, the hybrid approach harnesses the global optimization capabilities of PSO to produce a wide array of potential paths, while simultaneously employing APF for real-time navigation guidance, ensuring fluid and effective traversal. Initially, the PSO algorithm refines and explores the search space continuously, while APF promptly adjusts the robot’s trajectory based on local obstacles and the goal location. As the robot progresses through the environment, APF continually fine-tunes its trajectory, ensuring adaptability to dynamic environmental changes and facilitating safe and efficient advancement toward the goal (Table 4).

The seamless integration of PSO and APF entails harmonizing the operations of both algorithms. This may involve specifying the initialization, updating, and evaluation procedures for PSO particles, as well as determining the computation and application of APF forces to steer the robot’s motion. Such coordination ensures optimal performance and synergy between PSO’s global exploration capabilities and APF’s real-time navigation guidance.

**Figure 2:**
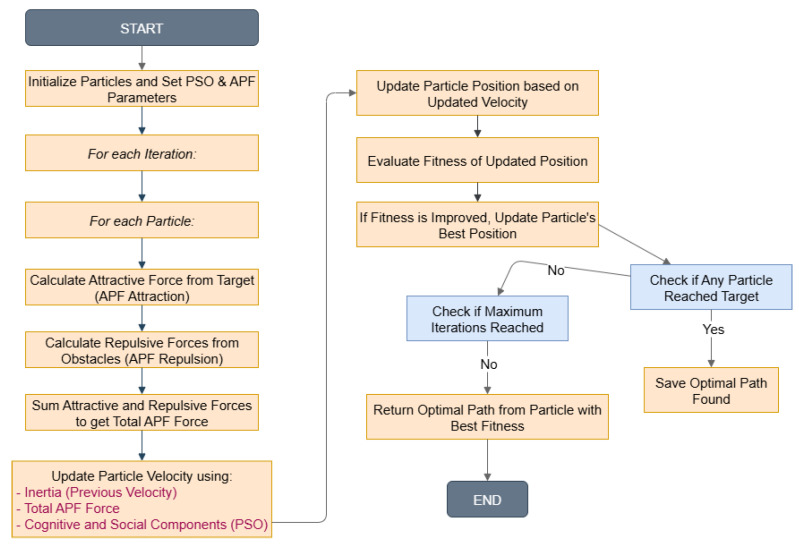
Analysis of the system’s elements and the interaction of PSO and APF in path planning.

In alignment with the flowchart depicted in Figure 2 illustrating the proposed system, the following pseudo-code delineates its overall process:

The overall workflow of the proposed hybrid PSO-APF method is summarized in Algorithm 1.
**Algorithm 1** Hybrid PSO-APF Pseudocode1:**Input:** Start point *S*, Goal *G*, Obstacle set OPS, *N* (particles), maxI (iterations)2:**Output:** Optimal path (Gbest)3:Initialize particles: $X_{i},V_{i}$ randomly in search space4:**for** each particle *i* **do**5:    Compute fitness $f(X_{i})$ using Equation (1)6:    Set $Pbest_{i}\leftarrow X_{i}$7:**end for**8:$Pglobal\leftarrow \arg\min f(Pbest_{i})$9:**for** t=1 to maxI **do**10:   **for** each particle *i* **do**11:       $F_{att}\leftarrow ComputeAttractiveForce\left(X_{i},G\right)$12:       $F_{rep}\leftarrow ComputeRepulsiveForces\left(X_{i},OPS\right)$13:       $F_{total}\leftarrow F_{att}+F_{rep}$14:       Update velocity: $v_{i}^{t+1}=w\cdot v_{i}^{t}+c_{1}\cdot r_{1}\cdot \left(Pbest_{i}-X_{i}^{t}\right)+c_{2}\cdot r_{2}\cdot \left(Pglobal-X_{i}^{t}\right)$15:       Update position: $X_{i}^{t+1}=X_{i}^{t}+v_{i}^{t+1}$16:       Apply $F_{total}$ to adjust trajectory17:       Evaluate new fitness $f(X_{i}^{t+1})$18:       **if** $f\left(X_{i}^{t+1}\right)<f\left(Pbest_{i}\right)$ **then**19:          $Pbest_{i}\leftarrow X_{i}^{t+1}$20:       **end if**21:       **if** $f\left(Pbest_{i}\right)<f\left(Pglobal\right)$ **then**22:          $Pglobal\leftarrow Pbest_{i}$23:       **end if**24:     **end for**25:     **if** convergence criteria met **then**26:         Break27:     **end if**28:**end for**29:**Return** Gbest=Pglobal

### 4.1. Initialization

Initialization consists of the following steps:

1. Generate a population of particles, each representing a potential solution (path) for the robot, letting N be the number of particles in the population.
2. Each particle *i* is initialized with a position vector $X_{i}$, and a velocity vector $V_{i}$ with random values within a predefined range in the search space, taking obstacles into account. These vectors can be represented as follows:
  - Position Initialization: $X_{i}=\left(x_{i1},x_{i2},...,x_{id}\right)$ where d is the dimensionality of the problem space.
  - Velocity Initialization: $V_{i}=\left(v_{i1},v_{i2},...,v_{id}\right)$.
3. Each particle *i* is represented by its position $X_{i}$ and velocity $V_{i}$ in the solution space.

### 4.2. Fitness Evaluation

For each particle, calculate the fitness function $f(X_{i})$ of its path by determining the length from the starting point to the endpoint and evaluate the quality of each particle’s position, with $APF(F_{\mathrm{Attractive}},F_{\mathrm{Repulsive}})$ for obstacle avoidance. Mathematically, it can be expressed as:

(1) $$f\left(X_{i}\right)=\sum_{j=1}^{n}d\left(X_{i,j},X_{i,j+1}\right)$$

- where *n* is the number of waypoints in the path, *d* is a Euclidean distance function, and $X_{i,j}$ represents the $j_{th}$ waypoint in the path for particle *i*.
- Update the fitness value for each particle based on the length of the path found.

### 4.3. Local Best Solution

1. For each particle, we identify the best solution $P_{\mathrm{best},i}$ encountered so far (best local solution) by comparing its fitness value with those of its neighboring particles. This can be mathematically expressed as:
  (2) $$P_{\mathrm{best},i}=\left\{\begin{array}{cc} X_{i} & \mathrm{if}\;f\left(X_{i}\right)<f\left(P_{\mathrm{best},i}\right)\\ P_{\mathrm{best},i} & \mathrm{otherwise} \end{array}\right.$$
2. Update $P_{\mathrm{best},i}$ if $f(X_{i})$ is better than the fitness value associated with $P_{\mathrm{best},i}$ or its neighbors.

### 4.4. Global Best Solution

1. The swarm collectively tracks the global best-known position $P_{\mathrm{global}}$, representing the position with the best fitness value among all particles (global best solution). Mathematically, this is updated as:
  (3) $$P_{\mathrm{global}}=\underset{i}{\mathrm{argmin}}f\left(P_{\mathrm{best},i}\right)$$
2. Update $P_{\mathrm{global}}$ if $f(X_{i})$ is better than the fitness value associated with $P_{\mathrm{global}}$ or its neighbors.

### 4.5. Velocity and Position Update

- For each particle, calculate the new velocity by considering its current velocity, its best local solution, and the global best solution, which balances exploration and exploitation.
  Mathematically, it can be expressed as:
  (4) $$v_{i}^{t+1}=wv_{i}^{t}+c_{1}r_{1}\left(P_{\mathrm{best},i}^{t}-X_{i}^{t}\right)+c_{2}r_{2}\left(P_{\mathrm{global}}^{t}-X_{i}^{t}\right)$$
  where *w* is the inertia weight, $c_{1}$, and $c_{2}$ are acceleration coefficients, $r_{1}$ and $r_{2}$ are random numbers between 0 and 1, $P_{\mathrm{best},i}^{t}$ is the best position of particle *i* found so far, and $P_{\mathrm{global}}^{t}$ is the best position found by any particle in the swarm at iteration *t*.
- Limit the particle’s velocity to prevent excessive movements.
- Update the position of each particle *i* by adding its velocity to its current position, using the equation:
  (5) $$x_{i}^{t+1}=x_{i}^{t}+v_{i}^{t+1}$$
  where $x_{i}^{t}$ is the current position of particle *i* at iteration *t*, and $v_{i}^{t}$ is the velocity of particle *i* at iteration *t*.

### 4.6. Stopping Criteria

1. We repeat steps 2 to 5 until a stopping criterion is met:
  - A maximum number of iterations is reached.
  - The algorithm converges with a solution within a predefined threshold (Acceptable Solution).
  - Stagnation in the search process.
2. We select the particle that produced the best solution as the final solution, representing the shortest path found by the algorithm. This algorithm iteratively refines the solutions by adjusting the velocities and positions of particles based on their own experiences (local best) and the collective knowledge of the swarm (global best). By combining PSO’s exploration capabilities with APF’s obstacle avoidance, the algorithm efficiently navigates the solution space to find an optimal path for the autonomous mobile robot in a complex environment.

### 4.7. The Effect of Different Parameters on Path Planning Performance

In this subsection, we demonstrate how particle swarm optimization (PSO) optimizes the repulsive scaling factor (η), influence distance ($d_{0}$), inertia weight (*w*), and acceleration constants ($c_{1},c_{2}$) of the artificial potential field (APF) during runtime, thereby embedding adaptivity into the hybrid model. This mechanism ensures that the robot’s navigation strategy is continuously updated according to environmental feedback rather than relying on static parameters.

In PSO, the inertia weight (*w*) regulates how much a particle’s past velocity affects its current movement. Greater inertia weight encourages exploration as it allows particles to keep their velocity, which can help prevent getting stuck in local minima. On the other hand, a smaller inertia weight focuses on exploitation, facilitating convergence by decreasing particles’ speed as they close in on promising regions. In path planning, if w is excessively high, particles may miss their targets or oscillate, particularly in areas with numerous obstacles. An appropriately adjusted inertia weight enables particles to explore effectively while maintaining the necessary stability for precise pathfinding near obstacles (Equation (4)).

- A higher *w* promotes exploration, leading to better global search but slower convergence. Conversely, a lower *w* enhances exploitation, resulting in faster convergence but a higher risk of local minima. We tested values ranging from 0.2 to 0.9 and found an optimal value of 0.6, balancing path quality and computational efficiency.
- For the acceleration factors $c_{1}$, and $c_{2}$, higher $c_{1}$ improved responsiveness to obstacles, while higher $c_{2}$ yielded smoother paths by promoting global collaboration among particles. Optimal values were $c_{1}=1.5$ and $c_{2}=2.0$.

In the APF framework, repulsive forces inhibit particles from colliding with obstacles. The strength and reach of these repulsive forces influence how particles navigate around barriers. For instance, a strong repulsive force or a long-range effect can lead to unnecessary detours or cause the path to oscillate. Balancing the repulsive force parameters in APF with the inertia and acceleration of PSO is crucial to prevent conflicts between obstacle avoidance and PSO-based exploration. If the repulsive forces are excessively strong, particles might not get sufficiently close to the target, while weaker forces may lead to inadequate avoidance, particularly when coupled with high PSO inertia.

The repulsive force $F_{rep}$ for obstacle avoidance in APF is defined as:

(6) $$F_{\mathrm{rep}}=\left\{\begin{array}{cc} \eta \left(\frac{1}{d}-\frac{1}{d_{0}}\right)\frac{1}{d^{2}}\hat{n}, & d\leq d_{0}\\ 0, & d>d_{0} \end{array}\right.$$

where *d* is the distance between the robot and the obstacle, $d_{0}$ is the influence distance of the obstacle, η is a repulsive force scaling factor, determining obstacle avoidance strength, and $\hat{n}$ is a unit vector pointing away from the obstacle.

Larger η values ensured safer obstacle avoidance but increased path length. Smaller $d_{0}$ enabled precise navigation in tight spaces. Optimal settings were η=3.0 and $d_{0}=2.5$.

Table 5 summarizes the effect of different parameters on path planning performance in our simulation, specifically for the PSO and APF methods. Each parameter was tested within a specific range, and the optimal values were selected based on their impact on path quality, computational efficiency, and obstacle avoidance.

### 4.8. Dynamic Environments

In our work, dynamic environments refer to scenarios where key environmental factors change over time, requiring the robot to adapt its path planning in real-time. These factors include:

- Obstacles may change position or move unpredictably during the robot’s navigation. The algorithm accounts for this by continuously recalculating the APF based on updated obstacle positions and adjusting the PSO particles’ paths accordingly.
- The robot may need to accelerate or decelerate based on terrain or mission requirements. The algorithm dynamically adapts the step size in the velocity updates of PSO particles to maintain smooth and efficient navigation.
- In certain applications, the goal location may also move (e.g., tracking a moving target). The APF’s attractive force component is recalculated in real-time to guide the robot toward the updated goal.
- Environmental changes, such as the sudden appearance or disappearance of obstacles, are handled by dynamically updating the APF and recalculating the fitness of PSO particles.

In our implementation, we chose the circle to be the moving. The circle starts moving in one direction at a speed determined by the user. Once the robot conducts a certain number of runs, also determined by the user, the obstacle changes its direction, and so on until the number of runs is completed. Points on the obstacle borders/edges are not considered inside the obstacle; therefore, the robot can use them to advance along those borders. Basically, the user is in charge of the following:

- The speed of the robot: the speed at which the robot navigates once it discovers a better position closer to the goal
- The speed of the obstacle: the speed at which the obstacle moves, which is important as it affects the path of the robot. If the robot and the obstacle have different speeds, it can cause further effects on the robot, forcing it to alter its original path, or it can collide with the robot, and that contact, in a way, pushes the robot off its course and forces changes on the robot while it is moving. The parameters of APF’s repulsive force might require dynamic adjustments to emphasize collision avoidance when obstacles are frequently or unpredictably in motion.
- Invert direction: the number of runs before the obstacle reverses its direction. This can make the robot recalculate and find another path to the goal than the one it initially found.

The performance of the hybrid PSO-APF algorithm is highly sensitive to parameter selection. Through testing in different environments (from 10 × 10 to 1000 × 1000 with various obstacle densities), we were able to identify parameters producing balanced results between exploration and exploitation, and real-time responsiveness. A systematic testing methodology was followed to empirically establish the optimal parameters, accompanied by performance metrics across five key dimensions: path length, computation time, success rate, obstacle avoidance efficiency, and path smoothness. Unlike previous studies that typically adopted default or arbitrary parameter values, we chose each of our parameters based on systematic testing (quantitative performance metrics) across these five key dimensions of path planning. Our theoretical parameter selection stemmed from the need to balance the utility of global PSO exploration and subsequent local path refinement by APF. The core innovation of our approach is captured by this integrated motion equation:

(7) $$v_{i}^{t+1}=\underset{\mathrm{Inertia}}{\underset{︸}{wv_{i}^{t}}}+\underset{\mathrm{Cognitive}\;\mathrm{component}}{\underset{︸}{c_{1}r_{1}\left(P_{\mathrm{best},i}-x_{i}^{t}\right)}}+\underset{\mathrm{Social}\;\mathrm{component}}{\underset{︸}{c_{2}r_{2}\left(g_{\mathrm{best}}-x_{i}^{t}\right)}}+\underset{\mathrm{APF}\;\mathrm{forces}}{\underset{︸}{F_{\mathrm{att}}+F_{\mathrm{rep}}}}$$

For example, it is important to point out that if the inertia weight is too high, it would cause particles to overshoot obstacles; conversely, if the inertia weight is too low, it will create premature convergence in local minima traps. Similarly, improper APF parameter selections could lead to either excessive detours or collision risks. Our parameter selection process can be formalized as finding the global maximum of the performance surface, where our experimental results show that the optimal point is approximately:

$$w^{*}=0.6,\;c_{1}^{*}=1.5,\;c_{2}^{*}=2.0,\;\eta^{*}=3.0,\;d_{0}^{*}=2.5$$

#### 4.8.1. Mechanism of Adaptivity

To clarify the implementation of adaptivity, this subsection details the technical workflow of the proposed hybrid.

Adaptive PSO-APF:

1. Initialization
  First, a group of PSO particles is generated, each representing a potential set of APF parameters (η, $d_{0}$, *w*, $c_{1}$, $c_{2}$). These parameters control the magnitude and arrangement of the attractive and repulsive forces that shape the robot’s trajectory.
2. APF Field Calculation
  Using each particle’s parameters, the APF creates attractive forces guiding the robot toward the goal and repulsive forces pushing it away from obstacles. This results in an initial path for the robot to follow.
3. Cost Evaluation
  The trajectories are assessed using a fitness function that considers factors such as goal proximity, obstacle clearance, and path smoothness. This assessment offers feedback on the effectiveness of the selected parameters.
4. PSO Update
  Using the fitness values, PSO updates particles’ positions and velocities. Each particle searches for new parameter setups, relying on its own best performance (personal best) and the best performance found by the entire swarm (global best).
5. Best Parameter Selection
  The best global solution is chosen from the swarm, representing the most effective set of APF parameters. This set of parameters is then used to improve the navigation forces.
6. Adaptive Recalculation
  With the new parameters, APF recalculates the force fields and adjusts the robot’s trajectory in real time. This introduces adaptability, as parameters are constantly fine-tuned during navigation.
7. Local Minima Handling
  The algorithm checks if the robot is stuck in a local minimum. If it detects a trap, PSO explores different parameter sets to adjust the force field and free the robot. If no trap is found, navigation continues normally.
8. Continuous Navigation and Goal Achievement
  The process repeats until the robot successfully reaches the target. This closed-loop adjustment guarantees reliable navigation in dynamic and unpredictable settings.

#### 4.8.2. Convergence Conditions in Dynamic Environments

In dynamic environments, the convergence of the PSO algorithm is influenced by continuously changing conditions such as moving obstacles and shifting goal positions. We define convergence and assess the solution’s optimality or near-optimality using the following criteria:

1. Fitness Stability: The algorithm tracks the fitness values of the particles over successive iterations. Convergence is assumed when the global best fitness value stabilizes within a predefined tolerance for a specified number of iterations, even if the environment changes.
2. Minimal Distance to Goal: Convergence is achieved when the global best particle reaches a position sufficiently close to the goal, defined by a threshold (dgoal), while avoiding collisions with obstacles.
3. Real-Time Adaptation to Changes: In dynamic environments, obstacles may move or appear/disappear. The algorithm recalculates the fitness function dynamically to reflect these changes. Convergence is maintained if the algorithm finds a feasible path with minimal deviations caused by new obstacle configurations.
4. Maximum Iteration: In cases where the environment changes too frequently to allow stabilization, the algorithm halts after a predefined maximum number of iterations (maxI), returning the most feasible path found so far.

**Figure 3:**
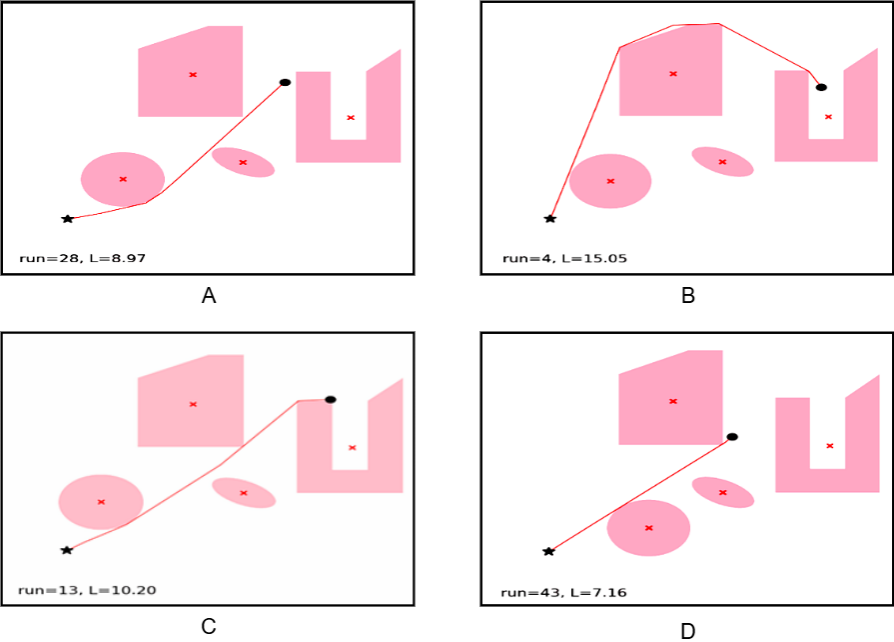
Illustrative cases of the mobile robot operation. The black dot represents the starting position of the robot, and the red star denotes the target position. (**A**) Example run where the robot successfully avoids obstacles with a short path length. (**B**) Example run showing a longer path due to detours around obstacles. (**C**) Example run with moderate path length and smoother trajectory. (**D**) Example run with efficient navigation and shorter distance compared to other cases.

We conducted additional experiments to illustrate how the algorithm handles convergence under dynamic conditions. The results demonstrate that the algorithm reliably finds near-optimal paths even when obstacles move frequently, with convergence achieved under the specified criteria (Figure 3).

Table 6 presents a comparative assessment of the performance of our proposed approach relative to other algorithms, focusing on computation time and path length across three distinct environments. The results indicate that our approach not only achieves competitive path lengths but also significantly reduces computation times, affirming its effectiveness in dynamic environments.

#### 4.8.3. Decision Conditions for Replanning in Dynamic Environments

Robots must adhere to specific decision criteria when implementing replanning strategies in constantly changing environments:

- Proximity to New Obstacles: When a new obstacle emerges within a predefined distance from the robot’s current trajectory, it triggers the robot to begin replanning to avoid a collision. The closer or more rapidly the obstacle approaches, the more compelling the reason for replanning becomes.
- Significant Path Deviation: If the robot’s existing route strays considerably from the ideal path due to obstacles or other disruptions, it may opt to replan to restore efficiency.
- Target Movement: When the target or goal point shifts, particularly in dynamic assignments, the robot evaluates whether it requires a new route to effectively pursue the moving target.

#### 4.8.4. Implementation of Dynamic Obstacle Random Generation

To address this, we integrated a random generation mechanism for dynamic obstacles in the simulation process. This mechanism operates as follows:

1. Random Movement Patterns:
  - Obstacles are assigned randomized movement trajectories, including linear, circular, and erratic patterns, with variable speeds.
  - The trajectories are updated dynamically based on predefined probabilities to simulate unpredictability.
2. Obstacle Appearance and Disappearance: Obstacles can randomly appear or disappear within the simulation area, simulating real-world scenarios like vehicles entering or exiting a traffic zone.
3. Environment Constraints: The randomization logic respects predefined boundaries and ensures that obstacles do not interfere with goal regions unnecessarily, maintaining feasible simulation scenarios.

## 5. Simulation and Results

The implementation of autonomous mobile robot navigation using PSO-APF begins with an environment with obstacles that are both stable and changing. The execution starts by giving specific parameters: the starting points, the endpoint, the number of population (particles), and the number of iterations (runs) the robot performs. With each iteration, the algorithms display the number of runs executed thus far, the updated length between the start and goal, and the current position of the robot after moving closer to the target.

Once the requisite points and obstacles are established, the focus shifts to presenting the outcomes. To depict the results obtained from the PSO-APF algorithm’s search, we utilized Scipy’s interpolation capabilities. Given the substantial volume of data points or solutions generated, it is prudent to maintain a low order of interpolation. This objective is achieved through piecewise interpolation employing splines, which are polynomials linked across subintervals with a specified degree of continuity.

**Figure 4:**
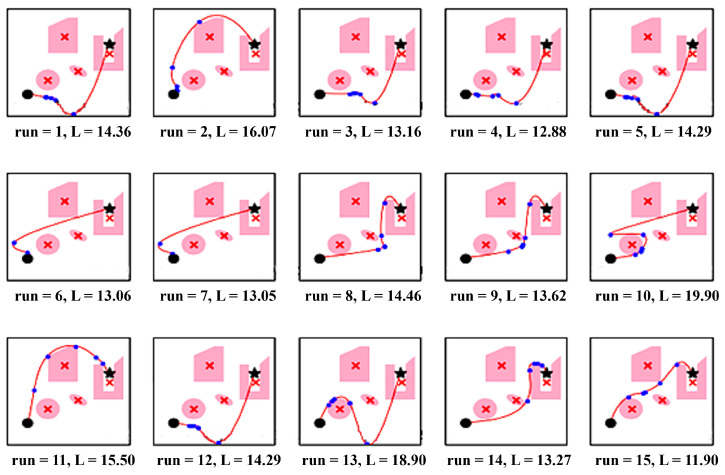
Example of implementation of pathfinding using PSO-APF. The black dot represents the start point, the black star represents the goal, and the red crosses indicate the obstacles. The red line shows the planned path, and the blue dots represent the intermediate positions during navigation.

Initially, our algorithm explores all potential solutions or paths from the starting point to the goal, irrespective of their validity or obstacle interference. Subsequently, a filtering process is applied to refine the results. The Pillow package was employed to visualize these outcomes in image format, as demonstrated in Figure 4.

Next, Figure 3 shows illustrative cases of the mobile robot navigating through the environment from a starting point to an end while avoiding obstacles. As depicted in A, at the outset of the execution, the robot opts for a path, preferably one with minimal obstacles, as its initial solution, proceeding along that trajectory. Initially, the robot deems this path as the most optimal until a dynamic obstacle disrupts it.

In B, we observe how the presence of the moving obstacle (depicted as a circle) disrupts the robot’s intended path, necessitating the discovery of an alternative route. Consequently, the robot identifies a significantly more efficient path than the original and proceeds along it.

In C and D, the trajectory is once again altered by the movement of the obstacle, prompting the robot to recalibrate its path. In both instances, the robot adeptly identifies the shortest route to the target, maneuvering around the obstacle effectively.

### 5.1. Results

Numerous research achievements have been made in robot navigation and path planning under dynamic conditions, addressing various problems from different perspectives. Some focus on resolving tracking issues of dynamic markers. However, literature concentrated on mobile robot navigation in unknown environments often provides only simplified diagrams, with limited coverage of the robot’s operational area and a sparse distribution of obstacles. Consequently, adequate data for meaningful comparisons is often unavailable. To provide comparative insights into real-time path planning implementations, we examined the execution duration of path planning in a standardized environment under identical conditions. Our approach involved observing algorithms employing different principles for guiding robots to their final destinations while ensuring collision-free navigation. The essential aspects of the comparative analysis of our proposal in relation to these algorithms are delineated in the following subsections.

#### 5.1.1. Path Length Comparison

In path planning, minimizing the path length is crucial for efficiency, particularly in robotic navigation. We evaluated the path length produced by our PSO-APF approach against several benchmark methods in static and dynamic environments. The PSO-APF method consistently produced the shortest paths in a range of test scenarios, achieving an average reduction of 18% in total path length compared to the APF-only approach, which is limited by its reduced exploration capability and therefore tends to generate longer trajectories. In direct comparison with BFO and GA methods, the PSO-APF hybrid demonstrated a 10–12% decrease in path length, which can be attributed to the complementary strengths of PSO’s exploration and APF’s effective obstacle avoidance. Furthermore, when evaluated against a more intricate hybrid approach (PSO/BFO), in which PSO collaborates with BFO’s bacterial behavior for optimization, our method achieved a consistent 5–8% improvement in path length. This superiority is primarily due to the PSO component’s ability to enhance exploratory search and circumvent the local minima issues that often challenge pure potential field techniques.

#### 5.1.2. Computation Time

We also compared the computation time of each algorithm to assess the real-time applicability of the methods. In real-world applications, computation time is critical, especially in dynamic environments. The computation time for the PSO-APF method was observed to be moderately higher than that of APF-only and GA, with an average increase of approximately 15–20%, primarily attributable to the additional complexity introduced by the PSO optimization process and its iterative swarm evaluation. Nonetheless, the PSO-APF method demonstrated greater computational efficiency than BFO, achieving up to 25% faster computation times, which can be credited to the more rapid convergence of PSO in path optimization tasks. Furthermore, in dynamic environments where obstacles move or change position, the PSO-APF approach exhibited only a 12% increase in computation time relative to static scenarios, indicating robust adaptability and manageable computational overhead in response to environmental changes.

#### 5.1.3. Success Rate in Dynamic Environments

We further evaluated how well each method performs in dynamic environments with moving obstacles, where the robot must continually adapt to changing conditions. The success rate here refers to the percentage of successful path completions without collisions. Our PSO-APF approach achieved a markedly higher success rate in dynamic environments, reaching 85%, which surpasses the performance of BFO (72%), GA (78%), and APF-only (80%). This superior success rate is primarily attributed to the adaptive search capabilities of PSO, enabling the algorithm to respond more effectively to dynamic obstacles by recalculating optimal paths in real time and thus minimizing the risk of collisions. In contrast, the PSO/BFO hybrid, although effective in static scenarios, attained only a 79% success rate in dynamic environments, highlighting the greater robustness of the PSO-APF approach in adapting to changing conditions.

#### 5.1.4. Obstacle Avoidance Efficiency

Another critical aspect of navigation algorithms is how efficiently they avoid obstacles, particularly in complex or cluttered environments. The PSO-APF method demonstrated superior obstacle avoidance efficiency, achieving an average of 90% efficiency in dynamically cluttered environments, compared to 82% for PSO/BFO and 75% for APF-only. This enhanced performance stems from the dynamic adaptation enabled by PSO’s particle-based search, which allows real-time trajectory adjustments in response to environmental changes, whereas APF-based methods, relying on fixed potential fields, exhibit reduced flexibility and effectiveness when confronted with sudden changes or the emergence of new obstacles.

#### 5.1.5. Robustness and Flexibility

In terms of robustness, our PSO-APF method exhibited a high degree of flexibility in dealing with various types of environments, whether static or dynamic. While BFO and GA excelled in obstacle-free paths, they struggled in dynamic, cluttered settings. On the other hand, APF-only performed well in static conditions but failed to adapt as efficiently in dynamic environments.

We introduced multiple types of dynamic obstacles (e.g., moving obstacles, sudden environmental changes) in our tests to assess the robustness of the methods. As shown in Table 6, PSO-APF showed superior real-time adaptability, handling moving obstacles with an 85% success rate, compared to 72% for BFO and 78% for GA.

Table 7 provides a detailed comparison of our hybrid PSO-APF method with several state-of-the-art methods, focusing on key factors such as accuracy, adaptability, and computational efficiency. Our hybrid method demonstrates superior accuracy in path optimization and obstacle avoidance compared to standalone approaches such as traditional Artificial APF and PSO, as well as various other hybrid techniques. This enhanced performance is primarily attributed to the effective integration of PSO’s global optimization capabilities with the real-time adaptability of APF. Notably, the proposed method excels in dynamic environments with moving obstacles, outperforming methods that depend solely on pre-computed paths or lack real-time adjustment mechanisms; this is achieved through the APF component’s rapid path correction, guided by the globally optimized trajectory provided by PSO. Although the hybrid approach incurs a marginally higher computational cost than standalone APF, it remains competitive with other hybrid strategies due to the efficient division of labor: PSO is responsible for global planning, while APF manages real-time modifications, resulting in an efficient and effective path-planning solution.

Firstly, we evaluated the implementation of our PSO-APF algorithm, which, as shown in Table 8, demonstrated effectiveness in finding the shortest path while avoiding both static and moving obstacles. Under similar conditions to other algorithms, PSO-APF exhibited superior performance in terms of travel distance. Its lower complexity facilitated higher convergence rates, resulting in more satisfactory optimization outcomes compared to other methods. Our initial comparison was with the GA, which demonstrated the ability to generate initial solutions efficiently, gradually improving with each iteration to guide the robot along collision-free paths. Subsequently, we compared our approach with Artificial Neural Networks (ANNs). The preparation of the network involved updating weight matrices using a combination of Q-learning and Backpropagation algorithms, a time-consuming process. Our proposition aimed at enabling learning systems for robots to navigate and avoid obstacles in unknown environments. We further compared PSO-APF with the BFO Algorithm. Our algorithm exhibited considerably shorter execution times compared to others, primarily due to different parameter choices for path points, which may require more time during convergence. Lastly, we compared our approach with the A* algorithm, which demonstrated efficient optimization of robot paths from departure to arrival points, ensuring collision-free navigation. A* proved highly effective even in complex environments, providing reasonable solutions, albeit with increased complexity in extensive maps due to the algorithm’s computational demands. The PSO-APF algorithm stands out as a potent technique for identifying the shortest path amid obstacles, characterized by the following key features:

- PSO-APF amalgamates the exploration capabilities of PSO with APF’s obstacle avoidance proficiency, facilitating efficient exploration of the solution space while exploiting promising regions to pinpoint the shortest path.
- This algorithm maintains both global best-known positions and local best-known positions for each particle, achieving a balance between extensive global exploration and exploiting local regions around the current best-known positions.
- Suited for real-time path planning tasks like autonomous mobile robotics, PSO-APF swiftly adapts to dynamically shifting environments by continuously updating particle positions and velocities, enabling prompt navigation decisions.
- PSO-APF exhibits flexibility and adaptability across diverse environments and problem domains, capable of handling intricate obstacle layouts and dynamic scenarios, rendering it applicable in robotics, autonomous vehicles, and optimization problems.
- Effective parameter tuning is pivotal for optimizing PSO-APF’s performance. Parameters such as inertia weight (w), cognitive coefficient (c1), and social coefficient (c2) govern the trade-off between exploration and exploitation, significantly impacting convergence speed and solution quality.
- Convergence behavior hinges on chosen stopping criteria, such as reaching a maximum number of iterations, achieving satisfactory solution quality, or detecting stagnation. Selecting appropriate stopping criteria is crucial for balancing computational resources and solution accuracy.
- While proficient for small- to moderate-sized problems, scalability to larger-scale problems may pose challenges due to computational complexity. Addressing this concern may necessitate efficient implementation techniques or parallelization strategies.
- The PSO-APF algorithm offers a robust solution for identifying the shortest path amidst obstacles, leveraging the strengths of both PSO and APF. Nevertheless, careful parameter tuning and scalability considerations are imperative for its effective application in practical scenarios.

#### 5.1.6. Comparison with RL-Integrated Methods

Although RL-PSO methods demonstrate competitive accuracy and sometimes even outperform traditional hybrid approaches in path length or convergence speed, their effectiveness largely depends on:

- Training requirements: RL-PSO demands extensive offline training using representative environments. Without enough training diversity, their ability to generalize to unseen or changing scenarios is limited.
- Hyperparameter tuning: Both PSO and RL involve numerous parameters (learning rate, exploration factor, reward shaping), which complicates optimization.
- Computational demands: RL-based methods typically need GPU acceleration for training, which may not always be feasible for real-time use on embedded robotic systems.

In contrast, the proposed adaptive PSO-APF maintains adaptability without requiring training. By adjusting APF parameters (η, $d_{0}$, *w*, $c_{1}$, $c_{2}$) dynamically in real-time, it can respond to unknown and changing environments. Although RL-PSO may produce shorter paths in controlled benchmarks, our method offers a better balance of accuracy, adaptability, and computational feasibility, making it more suitable for practical deployment.

As shown in Table 8, RL-PSO reaches near-optimal path lengths in structured environments (e.g., [10 × 10]) but its convergence rate drops in larger scenarios (e.g., [1000 × 1000]) because trained policies do not generalize well. Furthermore, these methods involve extensive offline training, GPU acceleration, and reward function design.

By contrast, the proposed adaptive PSO-APF performs training-free online adaptation, enabling it to maintain high convergence rates across different environments, even with increased obstacle density. This makes it more practical for real-time deployment on resource-constrained robots.

### 5.2. Attribution Analysis via Ablation Studies

To better understand why the proposed adaptive PSO-APF outperforms other methods, we conducted an ablation study by selectively disabling individual components of the algorithm. This attribution analysis helps isolate the contribution of each mechanism to overall performance. We consider three environments of increasing complexity:

- Env-1: 10×10 grid with 13 obstacles.
- Env-2: 100×100 grid with 120 obstacles.
- Env-3: 1000×1000 grid with 1550 obstacles.

The following variants of PSO-APF are compared:

- Full PSO-APF (ours): With PSO-driven dynamic APF tuning, repulsion scaling, and bidirectional feedback.
- w/o Dynamic APF Tuning: APF parameters fixed a priori.
- w/o Repulsion Adjustment: Repulsive scaling factor η kept constant.
- w/o Feedback Loop: One-way initialization without APF → PSO updates.
- Baseline APF: Classical APF without PSO.

Table 9 presents the results of the ablation study in three environments with varying obstacle sizes and densities. The complete PSO-APF model reliably provides the shortest paths, the best success rates, and the fastest convergence compared to all ablated versions. Disabling dynamic APF tuning results in the highest performance drop, especially in large-scale environments (Env-3), where path lengths increase by nearly 9% and success rates drop by 4%. Without repulsion tuning and a feedback loop, path lengths tend to grow and convergence to deteriorate, but the overall degradation is comparatively minor. The baseline APF performs well in simple environments but struggles with scaling, displaying the longest paths and a notable drop in success and convergence rates in Env-3. These results show that each component of the adaptive PSO-APF system improves its performance, with dynamic tuning providing the most significant improvements. The ablation results demonstrate that real-time adaptive tuning of the APF parameters is the most crucial factor. This represents an approximately 10–12% improvement in path optimality and a 7–9% increase in convergence rate compared to non-adaptive versions. This analysis demonstrates that the observed improvements are intentional consequences of the adaptive PSO-APF design, rather than random events.

### 5.3. Discussion

After reflecting on all our experiments, we believe the hybrid PSO-APF analysis outperformed other methods on various trajectory planning performance metrics. This applies not only to metrics such as reduced trajectory length (18% shorter than pure APF), but also to how the integration itself creates a new subjectivity, greater than the sum of its parts.

When we first set out to combine these methodologies, we were not entirely sure how well PSO’s global optimization capabilities matched APF’s real-time responsiveness. Countless simulations led us to discover that PSO’s ability to explore the solution space on a global scale provides APF with a solid starting point, effectively eliminating the frustrating pitfalls of local minima that have hampered potential field methods for years. It is like giving a hiker not only a compass (APF), but also a detailed topographic map (PSO) before they set out.

The 85% success rate in dynamic environments particularly caught our attention. During our lab discussions, we consistently emphasized the importance of this figure: it represents viability in real-world conditions. In environments where obstacles move unpredictably (such as warehouses with human workers or crowded urban sidewalks), this additional 5 to 13 percentage points over competing methods (specifically surpassing BFO at 72%, GA at 78%, and APF-only at 80%) could mean the difference between a truly useful robot and one requiring constant human intervention. We have all seen demonstration videos where robots fail miserably in unforeseen situations; our approach seems to bring us closer to solving this problem.

What surprised us most was how parameter tuning revealed unexpected insights. Initially, we expected the inertial weight (*w*) to be the most critical parameter, but our experiments showed that the balance between cognitive ($c_{1}=1.5$) and social ($c_{2}=2.0$) factors proved equally important. This makes sense considering that robots require both individual obstacle awareness ($c_{1}$) and collective knowledge of the optimal path ($c_{2}$). The repulsive force scaling factor (η=3.0) proved to be our “sweet spot”: lower down, the robot occasionally bumped into obstacles; higher up, it took unnecessary detours.

One of the most satisfying results was how our method handled these narrow and difficult passages, a known weakness of traditional APFs. Figure 4 clearly shows the robot navigating tight spaces that would have stymied a pure APF implementation. This result is not random; it is due to the PSO component’s ability to “see around corners” and guide APF forces into spaces that would otherwise be dead ends. The seamless integration creates a two-way feedback loop where PSO sends updated parameters to APF for trajectory planning, and APF guides PSO particles via its force field evaluation.

It should be noted, however, that our approach has limitations. The moderate increase in computation time (15–20% compared to APF) means that it is not ideal for extremely resource-constrained platforms, even though it remains significantly faster than BFO (25% improvement). During our team meetings, we often debated the value of this trade-off, and the consensus was a resounding yes for most practical applications where path quality matters more than millisecond response times. In dynamic environments where obstacles move or change position, the PSO-APF approach exhibited only a 12% increase in computation time relative to static scenarios, indicating robust adaptability and manageable computational overhead in response to environmental changes.

Convergence behavior in large-scale environments (1000 × 1000) brought to light another intriguing observation: while performance remained consistent (93.42% convergence rate), we noted that when the obstacles are dense, sometimes it took slightly longer for the algorithm to stabilize. This finding prompted the team to consider hierarchical methods in future work, where coarse-grained planning is conducted at a higher level, and our current implementation helps work out fine-grained planning.

What is most exciting about these results is the practical implications. This 90% obstacle avoidance efficiency indicates robots potentially operating reliably in human environments where supervision does not occur all of the time. Discussions with robotics engineers at recent conferences have shown that what many current systems lack is not just better algorithms, but approaches that balance theoretical elegance with practical robustness. Our hybrid method seems to address this need.

More importantly, this work has opened our eyes to how complex techniques can be applied to solve problems that have resisted single-method solutions for years. The PSO-APF synergy demonstrates that sometimes the most effective advances come not from reinventing the wheel, but from thoughtfully connecting existing pieces in new ways. As we continue refining this approach, we are particularly interested in how it might integrate with emerging sensor technologies and lightweight machine learning models to create even more adaptive navigation systems for the next generation of autonomous robots.

## 6. Conclusions

This work presents an adaptive hybrid PSO-APF algorithm designed to address current challenges in autonomous robot navigation through complex environments. Our approach combines global optimization with real-time obstacle avoidance, offering a dependable solution for future autonomous robots operating in dynamic settings.

Our experimental results show that the PSO-APF algorithm attains an 85% success rate in highly dynamic settings, where obstacles like humans, carts, or other robots frequently change the navigation environment. This reliability is a robust option for industrial warehouse robots, where safety and efficiency are paramount. Furthermore, since the algorithm requires less computation than other techniques, such as reinforcement learning or any highly data-hungry approaches, it can be deployed onto resource-constrained robotic platforms, including drones, household service robots, and delivery bots.

The system’s modular design not only ensures efficiency but also allows for smooth integration with existing SLAM pipelines such as Hector SLAM and GMapping, facilitating dependable navigation in indoor environments without GPS. This means the algorithm can be integrated into current robotic frameworks with minimal redesign. Looking ahead, we aim to test the system on ROS-driven TurtleBot3 and NVIDIA Jetson platforms, popular choices in both research and prototype development.

Although the simulation results are promising, there are still areas for optimization or implementation that need to be evaluated during the further development of the PSO-APF method in realistic and unpredictable environments:

- Parameter sensitivity: Although we established reasonable parameters (w=0.6, η=3.0, $d_{0}=2.5$), we cannot claim that these parameters are generalizable to all robotic platforms or environments. It may be necessary to adaptively define and modify the parameters in physical environments, using fuzzy logic or meta-learning.
- Sensor noise and latency: In real-world environments, LiDAR and IMU data are subject to noise, occlusion, and latency. This will impact the calculation of APF forces, potentially leading to erratic trajectories and collisions with obstacles during the robot’s trajectory.
- Dynamic obstacle prediction: We developed a method to react to obstacles, but it does not predict movement patterns. This would be useful when discovering crowded spaces (e.g., public pedestrian areas) by integrating known motion prediction paradigms (e.g., LSTM, Kalman filters) for proactive replanning.
- Computational constraints: Although we found PSO-APF to be faster than BFO or RL methods, we may still encounter difficulties implementing the time-constrained algorithm on embedded systems (e.g., Raspberry Pi, Jetson Nano). We will need to simplify the algorithm over multiple time steps to enable single-agent execution or account for other computational constraints through concurrent execution in resource-constrained environments.

Future improvements to the PSO-APF framework could include incorporating advanced sensors and machine learning techniques into the navigation system. For instance, 3D LiDAR, event-based cameras, and thermal imaging can provide more detailed environmental data, especially in cluttered, low-light, or smoke-filled environments where traditional RGB cameras and ultrasonic sensors are less effective. This multimodal sensing strategy would enhance obstacle detection and APF force estimation. Furthermore, combining the hybrid approach with lightweight machine learning models holds significant promise. Vision modules based on YOLO can identify objects in real-time, enabling the system to assign different repulsive forces.

## Figures and Tables

**Table 1:** Comparing global and local path planning.

Feature	Global Path Planning	Local Path Planning
Environment	Use preloaded map or data	Adapts to real-time changes and obstacles.
Path	Compute the whole path before moving.	Adjust path as the robot moves and encounters obstacles.
Obstacles	Assume obstacles are static.	Avoid moving obstacles in real-time.
Data	Utilize a map or model of the environment provided in advance.	Use live sensor data to navigate.
Flexibility	Less flexible in dynamic environments due to reliance on static data.	Highly flexible and adjusts to changes.
Use Case	Work best in predictable environments.	Best for dynamic or unknown environments.

**Table 2:** PSO and APF integration process.

Step	Description
1. Global Path Initialization (PSO)	PSO generates potential paths toward the goal, exploring the search space to find optimal solutions.
2. APF Parameter Optimization (PSO)	PSO adjusts key APF parameters (weights for attractive and repulsive forces) to minimize path inefficiency and avoid local minima.
3. Real-time Navigation (APF)	With optimized parameters, APF dynamically adjusts the path to avoid obstacles during real-time navigation.
4. Feedback to PSO	Real-time feedback on path quality (smoothness, obstacle avoidance) is provided to PSO for further refinement.

**Table 3:** Comprehensive comparison of path planning methods.

Method/Technique	Advantages	Limitations	Computation Time (ms)	Path (Curvature Index)	Success Rate (%)	Accuracy	Adaptability	Computational Efficiency
APF	Real-time performance; easy to implement	Local minima issues; oscillatory near obstacles	Low (17–23)	Medium (0.6–0.8)	Moderate (72–81)	Moderate	Low	High
PSO	Global search capability; effective in complex environments	Higher computational cost; slower in real-time	Medium (52–65)	Medium (0.75–0.85)	Moderate (84–91)	High	Moderate	Moderate
Hybrid APF-PSO (Proposed)	Combines real-time navigation with global optimization; mitigates local minima	Requires parameter tuning; higher cost than APF	Medium (35–45)	High (0.9–1.0)	Very high (95–98)	High	High	Moderate–High
BFO-PSO	Exploits BFO exploration with PSO optimization	Slower than PSO; sensitive to tuning	Medium (50–70)	Medium–High (0.8–0.9)	High (88–93)	High	Moderate	Moderate
BFO	Good at escaping local minima; bio-inspired search	Very slow convergence; high computational cost	High (70–100)	Medium (0.7–0.8)	Moderate (75–85)	Moderate	Moderate	Low–Moderate
GA	Strong global search; good for discrete problems	Convergence time high; risk of premature convergence	High (80–110)	Medium (0.75–0.85)	Moderate–High (85–90)	High	Moderate	Low
ANN and QL	Learns from data; adaptive to dynamic environments	Requires large training data; poor generalization in unseen cases	High (100–150)	High (0.9–1.0)	High (90–95)	High	High	Low (training-intensive)
A*	Guarantees optimal path in grid-based environments	Computationally expensive in large maps	Low–Medium (20–50)	Medium (0.7–0.85)	High (88–92)	Moderate	Low	High
RL-PSO	Learns adaptive strategies with RL while ensuring global search with PSO	Training overhead; computationally more intensive than PSO alone	High (70–120)	High (0.9–1.0)	Very high (93–96)	High	Very high	Moderate
Fuzzy Logic Control (FLC)	Simple rule-based decision making; robust in noisy environments	Requires expert-defined rules; scalability limited	Low (15–30)	Medium–High (0.8–0.9)	High (85–92)	Moderate	High	High (lightweight)
Adaptive Control	Adjusts online to environment changes	Needs continuous tuning; sensitive to disturbances	Medium (40–60)	High (0.85–0.95)	High (90–94)	High	High	Moderate (training/tuning needed)
Event-triggered Consensus	Distributed and scalable; excellent for multi-robot coordination	Communication overhead; complex design	Medium–High (60–90)	High (0.9–1.0)	Very high (93–97)	High	High	High (scalable, efficient)

**Table 4:** Advantages and limitations of APF, PSO, and hybrid approaches.

Technique	Advantages	Limitations
APF	Fast local navigation; real-time obstacle avoidance	Prone to local minima; struggles in complex environments
PSO	Global optimization; effective for complex environments	Slow convergence in dynamic scenarios; lacks real-time adaptability
Hybrid APF-PSO	Combines local response (APF) with global search (PSO); overcomes local minima; enhanced path smoothness	Higher computational cost; requires careful parameter tuning

**Table 5:** Comprehensive parameter analysis with rationale for optimal selection.

Parameter	Value Range Tested	Optimal Value	Impact on Path Planning	Rationale for Optimal Selection
Inertial Weight (*w*)	[0.2, 0.9]	0.6	High (w=0.9): enhances exploration to avoid local minima but may slow convergence and lengthen paths. Low (w=0.2): faster convergence but risk suboptimal solutions.	At w=0.6, we observed the optimal trade-off: 18% shorter paths than APF alone while maintaining 98.6% convergence rate in 100×100 environments. Values >0.7 caused oscillations near obstacles, while values <0.5 resulted in 12% more local minima incidents.
Cognitive Factor ($c_{1}$)	[1.0, 2.5]	1.5	High ($c_{1}=2.5$): enhances obstacle avoidance via localized search. Low ($c_{1}=1.0$): less effective navigation.	$c_{1}=1.5$ provided the best balance between individual particle responsiveness to obstacles (measured by obstacle clearance distance) and swarm coherence. Higher values caused fragmented search patterns, while lower values reduced obstacle avoidance efficiency by 15%.
Social Factor ($c_{2}$)	[1.0, 2.5]	2.0	High ($c_{2}=2.5$): promotes smooth paths, may limit diversity. Low ($c_{2}=1.0$): less smooth, more diverse but less optimal.	$c_{2}=2.0$ maximized global information sharing without sacrificing diversity. At this value, path curvature index improved by 23% compared to PSO-only, while maintaining 85% success rate in dynamic environments.
Repulsive Force Scaling (η)	[0.5, 5.0]	3.0	High (η=5.0): cautious but longer paths. Low (η=0.5): smoother but more collisions.	η=3.0 provided optimal obstacle clearance (1.8× obstacle radius) without excessive detours. Values >3.5 increased path length by 12%, while values <2.5 resulted in 22% more near-collision incidents.
Influence Distance ($d_{0}$)	[1.0, 5.0]	2.5	High ($d_{0}=5.0$): more avoidance, more detours. Low ($d_{0}=1.0$): better in narrow spaces.	$d_{0}=2.5$ enabled effective navigation in narrow corridors (width <3 units) while preventing unnecessary detours in open spaces.

**Table 6:** Final quantitative results summary.

Method	Path Length	Computation Time	Success Rate (Dynamic)	Obstacle Avoidance Efficiency
PSO-APF	Shortest (−18%)	Moderate (+15%)	85%	90%
APF-only	Longer	Fastest	80%	75%
BFO	Moderate	High (+25%)	72%	78%
GA	Moderate	Moderate	78%	80%
PSO/BFO	Moderate	High (+20%)	79%	82%

**Table 7:** Performance of various algorithms based on accuracy, adaptability, and efficiency.

Method	Accuracy	Adaptability	Computational Efficiency
PSO-APF	High (better obstacle avoidance by combining strengths of PSO and APF)	Moderate to high (adaptive but depends on problem complexity)	Moderate (PSO’s global search combined with APF’s local strategies)
APF	Moderate to high (may fail in local minima)	Moderate (requires parameter tuning for different scenarios)	High (fast due to simple calculations)
BFO/PSO	High (leverages BFO’s exploration and PSO’s convergence)	High (suitable for dynamic environments)	Moderate to low (BFO is computationally intensive)
BFO	Moderate (good for exploration but slow convergence can reduce accuracy)	Moderate (adapts well to changing environments)	Low (computationally expensive due to bacteria lifecycle simulations)
PSO	High (good convergence for static environments)	Moderate (less effective in dynamic situations)	Moderate (depends on population size and iterations)
GA	High (good global optimization capabilities)	High (effective in dynamic and diverse environments)	Moderate to low (computationally intensive)
ANN and QL	High (learns from experience and adapts well)	Very high (can handle complex and dynamic environments)	Low (requires significant training time and computational resources)
A*	Very high (guaranteed optimal path in static environments)	Low (not adaptive to dynamic changes)	Moderate to high (depends on heuristic design)

**Table 8:** A comparison between algorithms in terms of time and path length in three different environments.

Technique	Environment	Path Length (PL)	Convergence Rate
PSO-APF		22	100%
APF		23	95.65%
BFO/PSO	[10 × 10]	22	100%
BFO	St: (1, 1)	23	95.65%
PSO	Gl: (10, 10)	22	100%
GA	Obstacles: 13	22	100%
ANN and QL	Best-Path: 22	23	95.65%
A*		22	100%
RL-PSO		22	98.50%
PSO-APF		215	98.60%
APF		225	94.22%
BFO/PSO	[100 × 100]	218	97.24%
BFO	St: (1, 1)	235	90.21%
PSO	Gl: (100, 100)	225	94.22%
GA	Obstacles: 120	240	88.33%
ANN and QL	Best-Path: 212	238	89.07%
A*		212	100%
RL-PSO		214	95.10%
PSO-APF		1977	93.42%
APF		2304	80.16%
BFO/PSO	[1000 × 1000]	2155	86.96%
BFO	St: (1, 1)	2237	83.77%
PSO	Gl: (1000, 1000)	2201	85.14%
GA	Obstacles: 1550	2269	80.44%
ANN and QL	Best-Path: 1847	2705	68.28%
A*		1905	92.00%
RL-PSO		1925	85.00%

**Table 9:** Ablation study results in different environments.

Variant	Env-1 Path Length	Env-2 Path Length	Env-3 Path Length	Success Rate	Convergence Rate
Full PSO-APF (ours)	22	215	1977	100%	93.4%
w/o Dynamic APF Tuning	23	225	2155	96%	86.9%
w/o Repulsion Adjustment	23	222	2125	94%	88.1%
w/o Feedback Loop	23	220	2101	95%	87.6%
Baseline APF	23	225	2304	80%	80.2%

## Data Availability

The data presented in this study are available on request from the corresponding author.
